# Characterization of the Histopathologic Features in Patients in the Early and Late Phases of Cutaneous Leishmaniasis

**DOI:** 10.4269/ajtmh.16-0539

**Published:** 2017-03-08

**Authors:** Maíra G. Saldanha, Adriano Queiroz, Paulo Roberto L. Machado, Lucas P. de Carvalho, Phillip Scott, Edgar M. de Carvalho Filho, Sérgio Arruda

**Affiliations:** 1 Instituto Gonçalo Moniz, Fundação Oswaldo Cruz (FIOCRUZ), Salvador, Bahia, Brazil.; 2 Serviço de Imunologia, Complexo Hospitalar Universitário Professor Edgard Santos, Universidade Federal da Bahia, Salvador, Bahia, Brazil.; 3 Department of Pathobiology, School of Veterinary Medicine, University of Pennsylvania, Philadelphia, Pennsylvania.

## Abstract

Cutaneous leishmaniasis (CL), characterized by an ulcerated lesion, is the most common clinical form of human leishmaniasis. Before the ulcer develops, patients infected with *Leishmania* (*Viannia*) *braziliensis* present a small papule at the site of the sandfly bite, referred to as early cutaneous leishmaniasis (E-CL). Two to four weeks later the typical ulcer develops, which is considered here as late CL (L-CL). Although there is a great deal known about T-cell responses in patients with L-CL, there is little information about the in situ inflammatory response in E-CL. Histological sections of skin biopsies from 15 E-CL and 28 L-CL patients were stained by hematoxilin and eosin to measure the area infiltrated by cells, as well as tissue necrosis. *Leishmania braziliensis* amastigotes, CD4^+^, CD8^+^, CD20^+^, and CD68^+^ cells were identified and quantified by immunohistochemistry. The number of amastigotes in E-CL was higher than in L-CL, and the inflammation area was larger in classical ulcers than in E-CL. There was no relationship between the number of parasites and magnitude of the inflammation area, or with the lesion size. However, there was a direct correlation between the number of macrophages and the lesion size in E-CL, and between the number of macrophages and necrotic area throughout the course of the disease. These positive correlations suggest that macrophages are directly involved in the pathology of *L. braziliensis*–induced lesions.

## Introduction

Leishmaniasis is a broad term for anthropological zoonotic diseases caused by trypanosomes of the genus *Leishmania*. American tegumentary leishmaniasis (ATL) is characterized by a spectrum of clinical features, including asymptomatic infection, cutaneous leishmaniasis (CL), mucosal leishmaniasis, and disseminated leishmaniasis. CL is the main clinical form of the disease and it is characterized by one or more well-limited ulcers with raised borders, which develop at the site of the bite of infected sandfly. However, before the classical ulcer appears, patients often develop a lymphadenopathy in the lymph nodes draining the infection site, followed by the appearance of a nodule with a small superficial ulceration, which characterizes early CL (E-CL).^1^,^2^ The initial lesion increases in size and depth and between 4 and 6 weeks after the sandfly bite eventually forms an ulcer, the primary feature of late CL (L-CL). After the parasites are inoculated into the host, they interact with several different cell types, including macrophages, the major cell that harbors the parasite. Activation of macrophages by interferon (IFN)-γ^+^ produced by CD4^+^ T cells contribute to control parasite growth,^3^,^4^ whereas CD8^+^ T cells have been associated with pathology.^5^–^7^ Histopathological studies in ulcers of L-CL patients show an increase in inflammatory response, with the participation of T cells, B cells, plasma cells, macrophages, and the development of a granuloma.^8^–^11^ Although an intense lymphocyte proliferation and production of IFN-γ and tumor necrosis factor is induced on *Leishmania* antigen stimulation of peripheral blood mononuclear cells from patients with L-CL,^12^ in the preulcerative phase of the disease, lymphocyte proliferation, and cytokine production is lower than in patients with L-CL.^13^ Nevertheless, when compared with healthy subjects, E-CL patients exhibit an increase in the frequency of inflammatory or intermediate monocytes, produce higher levels of proinflammatory cytokines, and exhibit substantial transcriptional changes at the infection site.^2^,^14^ However, the histopathological features of E-CL have not been described. Therefore, in this study, we compared the histopathological features of biopsies from patients with E-CL and L-CL. We found that there are more parasites in biopsies from E-CL patients as compared with L-CL. Interestingly, there was no correlation between the number of parasites and the amount of inflammation or size of the lesions. However, there was a direct correlation between the number of macrophages with the area of necrosis and size of the ulcers.

## Methods

### Study design.

This is a cross-sectional study aimed to compare the histopathological features of skin biopsies from patients with E-CL and L-CL. Patients were attended in the Health Post of Corte de Pedra, Bahia, Brazil, reference center in the treatment of tegumentary leishmaniasis. All patients included in the study were adult. The study was carried out from April 2009 to May 2014. For every E-CL case selected and biopsied, two patients with L-CL were recruited by matching by age ±5 years. All patients denied previous history of CL and were clinically examined before therapy. After CL diagnosis, all were treated with intravenous glucantime 20 mg/kg/weight for 20 days as per the recommendation of Brazilian Ministry guidelines for CL. The clinical information used in this study was obtained from a public health clinic located in the rural countryside of the state of Bahia. Unfortunately, some data were incompletely recorded on the patient charts and, consequently, some analyses had data missing. The relevant sample size is consistently referenced in figures, tables, and descriptive texts.

### Biopsies and case definition.

E-CL is defined by the presence of a papular lesion occurring, according to patient reporting, within approximately 30 days of being bit by a phlebotomine.^1^ Patients with early cutaneous leishmaniasis seek medical attention due to the presence of a papular lesion associated with a painful regional lymphadenopathy. Ulcers typically appear 1–2 weeks after the appearance of papular lesions, which develop approximately 1–2 weeks after being bit by a sandfly.

Fifteen biopsies from E-CL and 28 from L-CL patients were analyzed. E-CL was defined by the presence of a papular lesion with less than 30 days of illness and a positive polymerase chain reaction (PCR) for *Leishmania braziliensis*. L-CL was defined by the presence of one ulcerated lesion with raised borders and a positive PCR for *L. braziliensis*. Only one skin fragment was obtained from each patient, and they were performed with a 4-mm punch. This fragment was divided in two parts, one for processing, and histological sections were stained with hematoxylin and eosin (H and E) and for immunohistochemical analysis, and other fragment was reserved in RNA for the quantitative PCR later.^15^

### Ethical considerations.

This study was approved by the Human Ethics Committee of the Research Center Gonçalo Moniz, Fiocruz, Bahia, protocol number 533.032/2014, and the Institutional Review Board of the Faculdade de Medicina da Bahia, Federal University of Bahia. A signed informed consent was obtained from all patients included in this study.

### Immunohistochemistry.

Skin biopsies were fixed in buffered formaldehyde and embedded in paraffin. Embedded tissue was cut in 5-μm thick sections, deparaffinized, and rehydrated. Antigens retrievals were performed using the Trilogy^™^ 1:100 (Cell Marque, Darmstadt, Hesse, Germany) at 96°C. Peroxidase activity was blocked with 3% hydrogen peroxide for 10 minutes and antibody nonspecific binding was blocked by the addition of serum-free protein (DakoCytomation, Carpinteria, CA) for 10 minutes. The slides were incubated at 25°C for 1 hour with the following monoclonal mouse anti-human antibodies and dilutions: anti-CD4, clone 4B12, 1:50 (DakoCytomation); anti-CD8, clone C8/144B, 1:200 (Cell Marque, Darmstadt); anti-CD20, clone L26, 1:200 (DakoCytomation); anti-CD68, clone M0814, 1:200 (DakoCytomation, Carpinteria, CA), and anti-*L. braziliensis* 1:1000 (in-house CPqGM Fiocruz).^16^ Peroxidase Kit and Rabbit mouse/horseradish peroxidase KP500 (Diagnostic BioSystems, Pleasanton, CA) and 3,3-diaminobenzidine tetrahydroxychloride was used to develop the antigen antibody reaction. All slides were counterstained by Harris hematoxylin, dehydrated, and mounted in Canada balsam and glass coverslips.

### Quantitative analysis.

The cells quantification was performed using an optical microscope BX51 (Olympus, Center Valley, PA) coupled with digital camera system Q5 (Olympus) and imaging software Image-Pro Plus (Media Cybernetics, Rockville, MD) to the micrograph of the slides was used. Ten random fields of each section with the respective antibodies were photographed using a magnifying power ×400. In each field, the number of positive cells was quantified using the counting feature of the semiautomatic software ImageJ 1.48v (National Institutes of Health, Bethesda, MD). Positivity was defined with the identification of cells that reacted with the chromogenic substrate.

### Morphometry of inflammation and necrosis areas.

The histological sections stained with H and E were scanned by an optical microscope BX61VS (Olympus, Center Valley, PA). The total extension of these sections as well as the areas of inflammatory infiltrate and necrosis was measured by Image J 1.48v (National Institutes of Health). The total length of the biopsy fragment and the sum of the areas of inflammation and necrosis are shown in mm^2^. The percentage (%) of inflammation and necrosis in the biopsies were calculated by dividing total extension of inflammation and necrosis in mm^2^ by the total extension of the biopsy fragment multiplied by 100.

### Statistical analysis.

For variables with normal distribution, we used the Student *t* test and post two-way analysis of variance test. For non-normal distribution, the nonparametric Mann–Whitney test was used. For correlations of normally distributed variables and non-normal, we used Pearson and Spearman tests, respectively. The strength of correlation was classified as: weak (*r* = 0.10–0.30), moderate (*r* = 0.40–0.60), and strong (*r* = 0.70–1). For comparison of the proportions, we used the Fisher's exact and χ^2^ test. Statistical analysis was performed using GraphPad Prism 1.5 (GraphPad Software, Inc., La Jolla, CA). The results were considered statistically significant for *P* < 0.05.

## Results

### Sociodemographic and clinical aspects.

**Figure 1.**
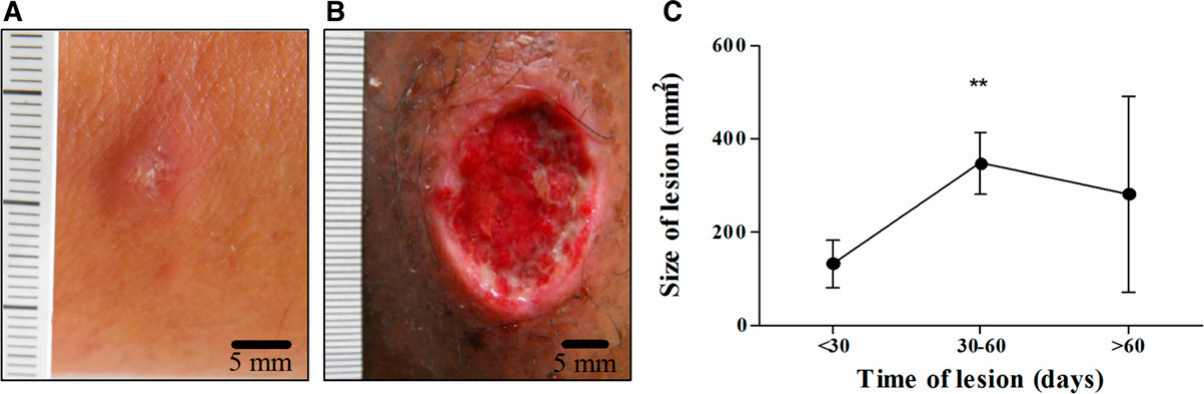
Differences between E-CL and L-CL lesions. (**A**) Papular lesion picture in E-CL compared with (**B**) L-CL ulcerated lesion caused by *Leishmania braziliensis.* (**C**) Size of skin lesions (mm^2^) according to duration of the disease (*N* = 43). ** *P* < 0.001 Kruskal–Wallis test. The bars represent standard error. To correlate the size of the lesion with the illness duration, we divided the sample into three groups. In the first group, we included patients with less than 30 days of illness; the second between 30 and 60 days; and the last over 60 days. E-CL = early cutaneous leishmaniasis; L-CL = late cutaneous leishmaniasis.

The sociodemographic and clinical features of the participants were stratified according to disease stage and are shown in Table 1. The age distribution was similar in the two groups. Males were more affected by the disease than women and the predominant localization of the lesions was in the lower limbs in both in E-CL and L-CL. The size of the *Leishmania* skin test was greater in patients with L-CL than in E-CL (*P* < 0.05) as well as the duration of the illness (*P* < 0.0001). Pictures of an E-CL lesion and a classical ulcer from L-CL are shown in Figure 1A and B

, respectively, and the size of lesions in different periods of the disease is shown in Figure 1C. The increase in the size and depth of the initial lesion occurred mainly in the first 30 days of the disease from 52.1 ± 11.1 up to 346.8 ± 60.0 mm^2^.

### Identification of amastigotes and relationship between *L. braziliensis* amastigotes with illness duration and lesion size.

**Figure 2.**
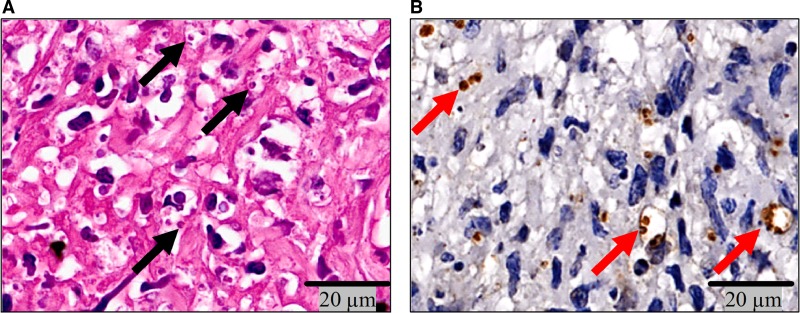
Amastigotes of *Leishmania braziliensis* in macrophages of E-CL. Amastigotes of *L. braziliensis* (**A**) in H and E (×40) (black arrows) and (**B**) immunostained with polyclonal antibody (red arrows) (×40). E-CL = early cutaneous leishmaniasis; L-CL = late cutaneous leishmaniasis; H and E = hematoxilin and eosin.

Confirming what was observed in H and E (Figure 2A

), tissue amastigotes were detected by immunohistochemistry using anti *L. braziliensis* IgG antibody (Figure 2B). In biopsies from E-CL analyzed by TEM, amastigotes were seen within macrophages (Figure 2C). Amastigotes were found mainly in the parasitophorous vacuole of macrophages, at the upper region of the dermis adjacent to the epidermis, as well as in areas of necrosis. No correlation was found between the number of amastigotes and areas of inflammation and necrosis (data not shown).

**Figure 3.**
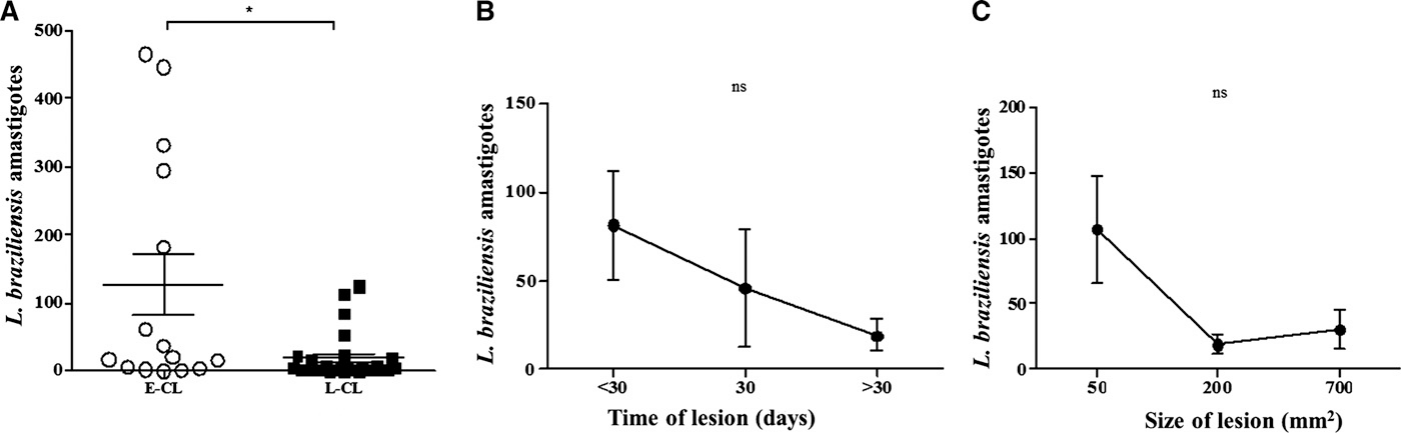
Number of amastigotes in E-CL and L-CL lesions. (**A**) Number of amastigotes of *Leishmania braziliensis* for 10 randomized fields of the histological sections from E-CL (*N* = 15) and L-CL (*N* = 28). * *P* < 0.01. The bars represent the mean and standard error. Statistical analysis was performed using the Student's *t* test. (**B**) Number of amastigotes according to the time of lesion and (**C**) according to lesion size. *P* = not significant (ns). Statistical analysis was performed using the Kruskal–Wallis test. E-CL = early cutaneous leishmaniasis; L-CL = late cutaneous leishmaniasis.

The relationship between the parasite load with phase of the disease, duration of illness, and lesion size is shown in Figure 3

. The parasitism was more intense in recent lesions than in classical ulcers. The number of parasites on 10 random fields under an optical microscope had a mean ± standard error of mean of 150 ± 56.6 for the E-CL and only 21 ± 8.0 in L-CL, *P* < 0.01 (Figure 3A). The relationship between the number of amastigotes and illness duration and size of the lesion is shown in Figure 3B and C. The number of amastigotes decreased with the illness duration and with the lesion size but did not reach statistical significance. The number of amastigotes was higher in papular lesions and decreased at the time of the ulceration indicating that ulcer formation is associated with a reduced parasite burden in the site.

### Inflammatory cell profile.

**Figure 4.**
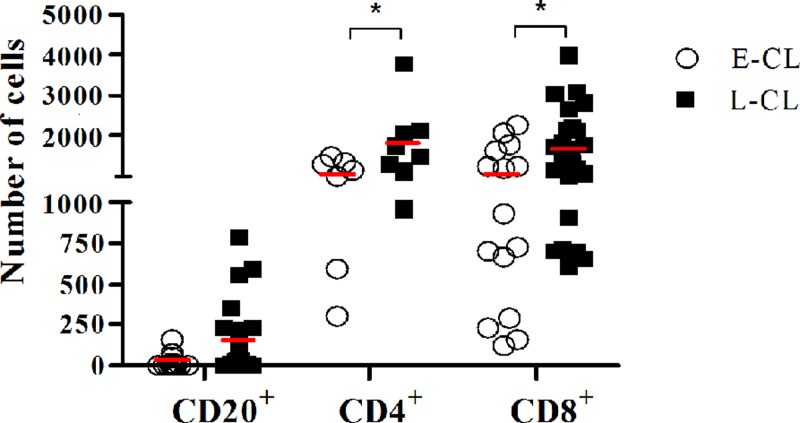
The number of B and T cells between E-CL and L-CL. Comparison of number of CD20^+^, CD4^+^, and CD8^+^ cells in 10?microscopic fields of E-CL and L-CL histological sections. The bars represent mean. *P < 0.05. Statistical analysis was performed using *t* student, post two-way ANOVA.

**Figure 5.**
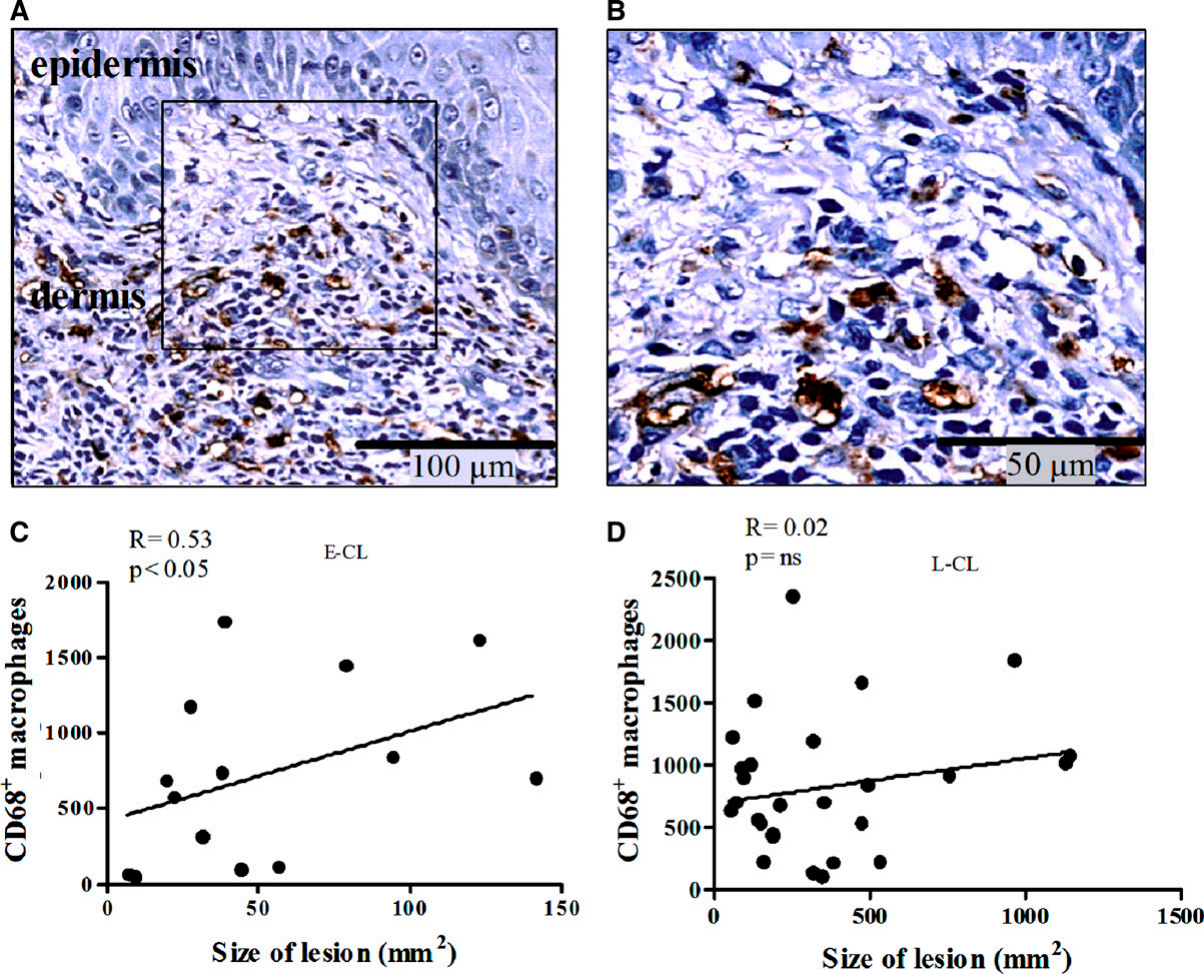
Correlation between CD68^+^ macrophages in histological section and correlated with size of lesion in E-CL and L-CL. (**A**) Macrophages immunostaining with monoclonal antibody anti-CD68 in E-CL (×10) (**B**) In detail CD68^+^ cells are grouped near epidermis and in close contact with other mononuclear cells (×20). Linear correlation between the number of CD68^+^ macrophages and size of the lesions in mm^2^ (**C**) in the E-CL (N = 14) and (**D**) L-CL (N = 28). Statistical analysis was performed using the Spearman correlation test. E-CL = early cutaneous leishmaniasis ; L-CL = late cutaneous leishmaniasis.

The inflammatory profile in both groups revealed a predominance of lymphocytes and CD68^+^ macrophages. CD4^+^ and CD8^+^ T lymphocytes were present throughout the sample from the dermal-epidermal junction and in granulomas. CD20^+^ B lymphocytes predominated in the middle portion of the dermis. The number of CD20^+^ cells did not differ significantly between early and late lesions, while there was an increase in the number of CD4^+^ and CD8^+^ T-cells in L-CL lesions compared with E-CL (Figure 4

). Macrophages were observed infiltrating the dermis at the junction of the epidermis and dermis up to hypodermis (Figure 5A, B

). Together with lymphocytes they were also present in areas of necrosis. Plasma cells, giant cells, and granulomas were seen. Vasculitis near the necrotic areas was also detected. Was observed a positive correlation observed between the number of macrophages and lesion size in E-CL (Figure 5C and D), there was no correlation between the frequency of CD4^+^ and CD8^+^ T cells and lesion size in E-CL (*P* > 0.05).

### Areas of inflammation and necrosis.

**Figure 6.**
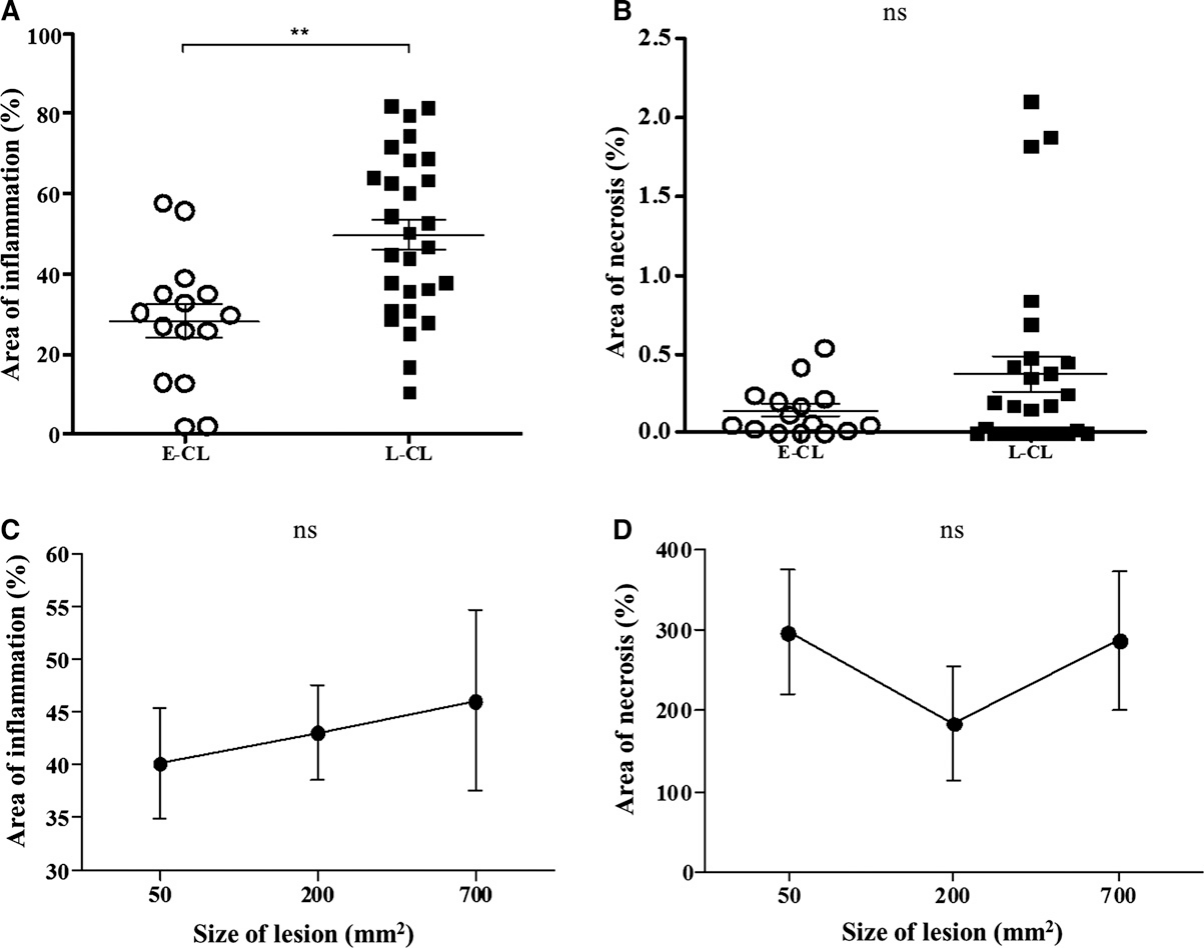
The area of inflammation and necrosis in E-CL and L-CL. (**A**) Evaluation of the area of inflammation to the total area of the histological section from E-CL and L-CL biopsies. ** *P* < 0.001. (**B**) Evaluation of area of necrosis to the total area of the histological section from E-CL and L-CL biopsies. *P* = not significant (ns). Statistical analysis was performed using the Student's *t* test, post-Mann–Whitney test. The bars represent mean and standard error. (**C**) Area of inflammation according to the size of lesion and (**D**) and area of necrosis according to the size of the lesion. *P* = ns. Statistical analysis was performed using Kruskal–Wallis test. E-CL = early cutaneous leishmaniasis (*N* = 15); L-CL = Late cutaneous leishmaniasis (*N* = 28).

The area of inflammation and necrosis in E-CL and L-CL and the relationship between them with the size of the lesions are shown in Figure 6

. As might be expected, the area of inflammation in E-CL (28.4% ± 4.2) was lower than in L-CL (49.8% ± 3.8) (Figure 6A). Lytic necrosis was seen in small areas. There was no difference between area of necrosis in both groups analyzed (Figure 6B). There was also no correlation between the percentage of the areas of inflammation and necrosis in both groups (data not shown).

**Figure 7.**
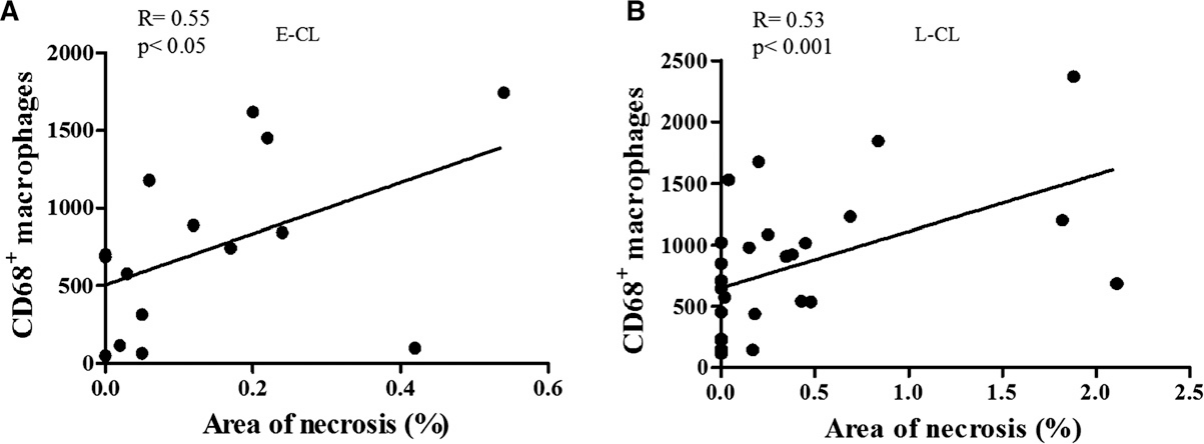
Number of macrophages CD68^+^ according to area of necrosis. Linear correlation between the number of CD68^+^ macrophages and area of necrosis in mm^2^ (**A**) in the E-CL (*N* = 15) and (**B**) in the L-CL (*N* = 28). Statistical analysis was performed using the Spearman test. E-CL = early cutaneous leishmaniasis; L-CL = late cutaneous leishmaniasis.

Although there was no association between CD68^+^ cells with the area of inflammation, there was a positive and significant correlation between the frequency of CD20^+^ B cells and the inflammation (*R* = 0.51; *P* < 0.05) (data not shown). There was a direct correlation between the number of CD68^+^ cells and the area of necrosis in both phases of the disease (E-CL and L-CL) (Figure 7A and B

). Finally, we found a direct correlation between the frequency of T and B cells and inflammation, but no correlation between the number of CD4^+^ and CD8^+^ T cells with the number of amastigotes, the lesion size, or the area of necrosis.

## Discussion

The cutaneous ulcer with raised borders is the most common presentation of CL occurring in more than 90% of patients infected with *Leishmania Viannia braziliensis*. However, before the ulcer appears in the skin, patients often present with a lymphadenopathy usually with a mild skin desquamation at the site of the parasite inoculation. This is followed by the development of a nodule that leads to a detectable ulcer. This initial phase of the disease characterizes E-CL. Although the immunopathology of the classical ulcerated lesion, a feature of L-CL, is well described, there is a lack of information about the histopathology in E-CL. In this study, we showed that parasite load is higher in E-CL than L-CL, but the parasite load is neither associated with the size of the ulcers or with ulcer development. Alternatively, there was a direct correlation between the frequency of macrophages with the area of necrosis and ulcer size.

Different from CL caused by other *Leishmania* species in which parasites are easily found in the skin lesion,^17^ in *L. braziliensis* ulcers, amastigotes are scarce or even absent under light microscopy examination. Here we showed that the amount of amastigotes was higher in E-CL than in L-CL biopsies. As expected, amastigotes were predominantly found inside macrophages, but parasites were also found outside of these cells and in the collagen of dermis. The absence of association between the parasite load and the area of inflammation, area of necrosis, and size of the ulcer suggest that the parasite load does not play a direct role in lesion development. This finding is in agreement with a previous report of a disparity between parasite numbers and the intensity of the inflammatory and necrotic events in L-CL.^18^ As macrophages are the main cells responsible for *Leishmania* killing, one could expect an inverse correlation between the numbers of CD68^+^ cells and amastigotes. Interestingly, we found a direct correlation between the frequency of CD68^+^ cells and number of amastigotes and there was an association between macrophages number and the area of necrosis in both E-CL and L-CL and between majority and size of the ulcer and CL. It has been shown that macrophages from CL patients exhibit an enhanced inflammatory profile, but are less able to kill *Leishmania*.^19^–^21^ Therefore, it is likely that parasite survival and leishmania antigen derived from dead parasites stimulate the adaptive immune response, thereby enhancing the inflammatory reaction.

The role of CD4^+^ and CD8^+^ T cells in the pathogenesis of L-CL is well documented. Although the T-cell response is important to prevent parasite dissemination, an exaggerated inflammatory response is associated with pathology.^22^,^23^ We have previously shown a direct correlation between the frequency of CD4^+^ T cells expressing IFN and TNF, and CD4^+^ T cells expressing lymphocyte activation markers with the lesion size.^22^,^24^ CD8^+^ T cells also play a role in the pathology.^4^,^25^ Although there was no association between CD8^+^ T cells expressing granzyme and the area of inflammation in E-CL, there was a correlation between the frequency of CD8^+^ T cells expressing granzyme and the intensity of the inflammatory reaction in L-CL.^26^ Moreover, although CD8^+^ T cells kill *L. braziliensis*–infected cells, they have an impairment in parasite killing.^4^,^5^,^23^ The inflammatory reaction in both E-CL and L-CL is composed of CD68^+^, CD4^+^ and CD8^+^ T cells as well as B cells. As T cells and macrophages are responsible for the granuloma formation, the role of these cells in the pathogenesis of CL has been well studied. In contrast, little emphasis is given for the role of B cells in the pathogenesis of *L. braziliensis* infection. B cells are found in high frequency in tissue of CL patients.^27^,^28^ Here we showed that CD20^+^ B cells are also observed in E-CL and the correlation between the frequency of B cells and the inflammation area pointed out the need for future studies to determine the participation of antibodies in the control of the infection or in the pathology of CL.

Although the area of inflammation was greater in L-CL than in E-CL biopsies, there was no difference between the area of necrosis in the two phases of the disease. The size of the lesion directly correlated with illness duration and similarly the area of inflammation was greater in L-CL than in E-CL. However, there was no correlation between inflammation and size of the lesions and there was also no correlation between the inflammatory and areas of necrosis. Necrosis seen in our study was small and focal in the majority of the biopsies. The pathogenic mechanisms leading to necrosis during CL are not well elucidated. Likely, this process is multifactorial, including vessel obliteration induced by vasculitis,^1^,^29^ killing of macrophages and epithelial cells expressing *Leishmania* antigen, and tissue injury by the inflammatory response.^30^–^32^ It is known that metalloproteinase (MMP) genes are highly expressed in the tissue of CL patients and that monocytes secrete high levels of MMP-9.^33^–^35^ MMP expression by macrophages may explain our findings of a direct correlation between macrophages and the area of necrosis in CL throughout the disease. Additional studies should be performed to identify if a programmed necrosis by activity of protein kinase RIPK3 is occurring.^36^

We recognize that the limited sample size in our study may have prevented a better correlation between some variables and inflammation or the area of necrosis. Although longitudinal studies using biopsies of the same patients in the two phases of the disease could help to better understand the dynamics of the immunopathology, this is not possible as patients are treated upon diagnosis. Despite a few limitations, our immunopathologic study comparing biopsies from patients with E-CL versus L-CL contributes to the understanding of host and parasite factors in the pathogenesis of *L. braziliensis* and emphasizes the participation of macrophages in the development of CL ulcers.

We have previously shown that although macrophages from patients with CL have an impairment in *Leishmania* killing, they produce high levels of proinflammatory cytokines, such as TNF and the chemokines CXCL9 and CXCL10.^19^ These molecules contribute to necrosis and cell recruitment to the site of infection. Monocytes and macrophages are heterogeneous subpopulations, with killing, inflammatory, and regulatory profiles.^34^ Previous studies have shown a high frequency of monocytes with inflammatory profile in E-CL,^14^ and there is a direct correlation between the frequency of monocytes expressing toll-like receptor^9^ with ulcer size in CL.^37^ Moreover, no production rather than protection is associated with pathology in *L. braziliensis* infection.^38^ It is clear that monocytes and macrophages have also protective function killing intracellular pathogens. However, while *Leishmania* killing is mediated by classical monocytes, secretion of proinflammatory cytokine is produced mainly by the inflammatory monocytes.^3^,^14^ Therefore, the increase in proinflammatory monocytes in CL and even in E-CL may explain the intense inflammatory reaction and parasite persistence. Because of the plasticity of monocyte population and limited numbers of cells obtained in the biopsies, studies on monocyte subsets in tissue are limited. However, our documentation that macrophages number correlates with the necrosis area and lesion size in E-CL indicates that in addition of CD4^+^ and CD8^+^ T cells, macrophages play a role in ulcer development in CL due to *L. braziliensis*.

ATL is one of the best examples about the tenuous line that separate protection from pathology. Here, although we showed that parasite burden was not associated with inflammation and ulcer size, it was clear that pathology due to inflammation and necrosis occurred due to an attempt of the host to eliminate parasites. Different from many other infectious diseases in which early therapy is associated with a high rate of cure and acceleration of the healing time, patients with E-CL have a high rate of failure to antimony therapy in comparison with L-CL. The documentation of high number of amastigotes early in the infection and a progressive inflammatory reaction with illness duration indicate that in addition to parasite killing, a down modulation of the inflammatory reaction should be attempted in the treatment of patients with E-CL.

## Figures and Tables

**Table 1:** Sociodemographic and clinical data of patients with E-CL and L-CL

	E-CL (*N* = 15)	L-CL (*N* = 28)	*P*
Age (years)*	36.9 ± 3.0	39.3 ± 2.9	ns‡
Gender *n* (%)			ns§
Female	5 (33.3)	6 (21.4)	
Male	10 (66.7)	22 (78.6)	
Lesion site *n* (%)			ns¶
Lower limbs	13 (86.7)	24 (85.7)	
Upper limbs	1 (6.7)	3 (10.7)	
Others	1 (6.6)	1 (3.6)	
Lymphadenopaty *n* (%)†			ns¶
Yes	14 (93.3)	17 (63.0)	
No	1 (6.7)	10 (37.0)	
Skin test (mm^2^)*	129.5 ± 15.4	196.9 ± 22.8	< 0.05‡
Size of lesion (mm^2^)*¶	52.1 ± 11.1	346.8 ± 60.0	< 0.0001‡
Time of lesion (days)*	17.4 ± 1.6	40.6 ± 4.4	< 0.0001‡

E-CL = early cutaneous leishmaniasis; L-CL = late cutaneous leishmaniasis; ns = not significant.

* Mean ± SEM (standard error of the mean.

† Data missing: one 1ess lymphadenopathy in L-CL and one less size of lesion in E-CL measurements.

‡ Student's *t* test.

§ Chi-squared test.

¶ Fisher's exact test.
